# Adapting cognitive behavioural therapy for adolescents with psychosis: insights from the Managing Adolescent first episode in psychosis study (MAPS)

**DOI:** 10.1080/17522439.2021.2001561

**Published:** 2022-01-03

**Authors:** Amy Langman-Levy, Louise Johns, Jasper Palmier-Claus, Catarina Sacadura, Ann Steele, Amanda Larkin, Elizabeth Murphy, Samantha Bowe, Anthony Morrison

**Affiliations:** a Early Intervention in Psychosis Service, Warneford Hospital, Oxford Health NHS Foundation Trust, Oxford, UK; b Department of Psychiatry, Medical Sciences Division, University of Oxford, Oxford, UK; c Spectrum Centre for Mental Health Research, Division of Health Research, Lancaster University, Lancaster, UK; d Research and Development Department, Lancashire and South Cumbria NHS Foundation Trust, Preston, UK; e Research and Development Department, Sussex Partnership NHS Foundation Trust, Sussex, UK; f Psychosis Research Unit, Greater Manchester Health NHS Foundation Trust, Manchester, UK; g Division of Psychology and Mental Health, Faculty of Biology, Medicine and Health, University of Manchester, Manchester, UK

**Keywords:** Adolescence, early onset, first episode, psychosis, cognitive behaviour therapy, CBT

## Abstract

**Background:**

Onset of psychosis commonly occurs in adolescence, and long-term prognosis can be poor. There is growing evidence, largely from adult cohorts, that Cognitive Behavioural Therapy for Psychosis (CBTp) and Family Interventions (FI) can play a role in managing symptoms and difficulties associated with psychosis. However, adolescents have distinct developmental needs that likely impact their engagement and response to talking therapy. There is limited guidance on adapting CBTp to meet the clinical needs of under-eighteens experiencing psychosis.

**Method:**

This educational clinical practice article details learnings from therapists and supervisors working with young people (aged 14–18 years) with psychosis during the Managing Adolescent first-episode Psychosis: a feasibility Study (MAPS) randomised clinical treatment trial, supplemented by findings from nested qualitative interviews with young people receiving CBTp.

**Results:**

Suggested are given for tailoring CBTp assessment, formulation and interventions to meet the developmental and clinical needs of adolescents with psychosis. Developmentally appropriate techniques and resources described.

**Conclusions:**

Early indications from MAPS study indicate this developmentally tailored approach is an acceptable, safe and helpful treatment for young people with psychosis. Further research is needed to develop empirically grounded and evaluated CBTp for adolescents.

## Introduction

Onset of psychosis typically occurs in adolescence and early adulthood (Jones, 2013). The period of adolescence confers considerable vulnerability to mental health difficulties. Adolescence is characterised by substantial psychological, biological, and social developments formative in navigating transition to adulthood. These developmental advances are associated with riskier impulsive behaviours, increased self-consciousness, heightened regard for peer acceptance, and sleep disturbance (Blakemore, 2019); circumstances under which paranoia and psychosis can flourish.

Early-onset psychosis (EOP) refers to a first episode of psychosis developing before the age of 18 years. Estimates of incidence rates in the UK are 5.9 per 100,000 people (Boeing et al., 2007). Evidence suggests that the earlier psychosis presents, the greater severity of symptoms and poorer prognosis with worse functional outcomes and greater numbers of psychiatric hospitalisation and relapses (Díaz-Caneja et al., 2015). In many cases, psychosis follows a chronic and relapsing course with long-term psycho-social impairment and poses unique challenges to family members and carers (e.g. Patel et al., 2014). Psychiatric co-morbidities are common including anxiety, depression, substance abuse, and post-traumatic stress disorder (Buckley et al., 2009).

Psychosis can be a severely debilitating mental health difficulty that requires timely specialist intervention (National Collaborating Centre for Mental Health and the National Institute for Health and Care Excellence, 2016). Skilled clinical judgment is required to tailor treatment for the complex and emerging experiences of adolescents with first-episode psychosis. Pathways from Child and Adolescent Mental Health Services (CAMHS) to specialist Early Intervention for Psychosis Services (EIPS) are often challenging (Singh et al., 2015), with potentially limited psychosis expertise in generic CAMHS and modest developmental expertise within EIPS.

Clinical guidelines recommend psychological interventions should be offered in conjunction with antipsychotic medication (National Collaborating Centre for Mental Health [NCCMH], 2014; updated 2016). However, evidence for the effectiveness or acceptability of psychological therapies for adolescents with psychosis is limited (NICE Clinical Guideline 155, National Institute for Health and Clinical Excellence [NICE], 2013). Whilst preliminary trials and case studies of young people (YP) with distressing ”unusual experiences” indicate that Cognitive Behavioural Therapy for psychosis (CBTp) and family intervention (FI) can improve psycho-social functioning (e.g. Browning et al., 2013; Jolley et al., 2018), further evaluation is required (Haddock et al., 2006). Adaptations may be required to address YP’s reluctance to seek help and engage with psychological treatment (Radez et al., 2020), and sceptical preconceptions held by clinicians about the capacity of this population to engage in a structured talking therapy (Byrne et al., 2020; Haddock et al., 2006).

Despite the wealth of clinician expertise and progressive research underway to refine interventions and models of delivery for treating psychosis in adolescents (e.g. Browning et al., 2013; Fowler et al., 2019), many clinicians may benefit from further guidance to advance provision and outcomes of CBTp for adolescents. The aim of this empirically grounded clinical practice guidance paper is to provide suggestions to inform delivery of CBT for adolescent first-episode psychosis (FEP) and may therefore be of value to readers working with YP experiencing first-episode psychosis.

## Method

This paper aims to disseminate clinical practice learnings to help tailor CBTp to meet the developmental needs of adolescence with psychosis. Learnings are drawn from a group of highly specialist trial therapists and expert supervisors delivering CBT to adolescents (aged 14–18 years) with first-episode psychosis as part of the Managing Adolescent first-episode Psychosis: a feasibility Study (MAPS) randomised clinical treatment trial conducted in England (Morrison et al., 2021, 2020),^1^ supported by findings from nested qualitative interviews investigating treatment views of YP and family members who received this psychological treatment (For further information, see Byrne et al., 2020).

Within the MAPS study, trial therapists delivered CBTp to 39 adolescents randomly allocated to receive psychological intervention either alone or alongside anti-psychotic medication. Psychological intervention used a specific cognitive model guided by “a manualised treatment protocol to guide delivery of evidence-based cognitive therapy for people with distressing psychosis” (Morrison, 2017). This allowed an individualised approach incorporating a process of assessment and psychological formulation of problems and goals, which guided specific interventions focusing on developmental and maintenance factors. To adapt CBTp for adolescents, trial therapists drew initiatives from assertive outreach approaches developed from youth work principles (e.g. Fowler et al., 2019), and neuro-developmental theories of adolescence (e.g. Blakemore, 2019).

Trial therapists offered up to 26 weekly sessions of individual CBTp (medium attended 14.5) over a 6-month treatment period with 4 optional booster sessions in the subsequent 6 months. Up to 6 optional sessions of FI were offered, generally delivered by the same therapist, in tandem to match the pacing and content of CBT sessions. Fidelity to protocol was ensured through session-content adherence checklists, regular expert supervision and competence rating of recorded sessions using the Cognitive Therapy Scale–Revised (Blackburn et al., 2001).

## Results

This article aims to provide practical, accessible guidance on tailoring CBTp to optimise engagement with adolescents with psychosis. Suggestions for techniques and resources to accommodate CBTp assessment, formulation, and interventions to meet the specific developmental and clinical needs of adolescents experiencing first-episode psychosis are detailed. Key socio-cognitive developmental and systemic factors common to adolescents that may impact engagement and progress with CBTp are described, along with suggested adaptations for each phase of CBTp delivery as follows: phase 1: Assessment and engagement (including creating focus, building engagement and involving others, monitoring and motivating change, and potential challenges to working with adolescence); phase 2: Formulating maintenance factors and change strategies; phase 3: Longitudinal formulations and CBTp; phase 4: Consolidation and promoting “Staying Well”. These adaptations can be applied flexibly using clinical judgment, with the aim to build engagement, motivate change, and overcome challenges specific to delivering CBTp with adolescents experiencing psychosis.

### CBTp phase 1: assessment and engagement

CBTp commences with assessment of presenting difficulties to identify precipitating and predisposing factors, triggers, appraisals, emotions, and responses. It is key to incorporate understanding of the broader socio-demography, cultural systems and generational subcultures that interact and impact a young person’s needs and presenting problems. Consider the nature and quality of family and peer circumstances, as well as social, educational, or work environments to identify contextual factors that exacerbate or ameliorate symptoms or hinder therapeutic engagement and progress. This holistic view helps inform selection of goals and change strategies.

#### Creating focus: devising a problem list and generating goals

To create therapeutic focus establish a problem list and person-centred goals (ideally by the third session). Resolution of psychotic symptoms may not be the primary focus of CBTp, particularly if already reduced by medication. It is appropriate to select goals unrelated to psychosis if this focus promotes gains in a young person’s mental health. Within the MAPS trial, YP frequently identified co-morbid mental health and personal problems as their therapeutic objectives (e.g. confidence building, managing peer relationships and social isolation, boosting concentration, substance misuse, self-harm). If non-psychotic difficulties, such as panic attacks, were prioritised the therapist would utilise the relevant evidenced-based cognitive model to inform treatment.

**Figure 1.**
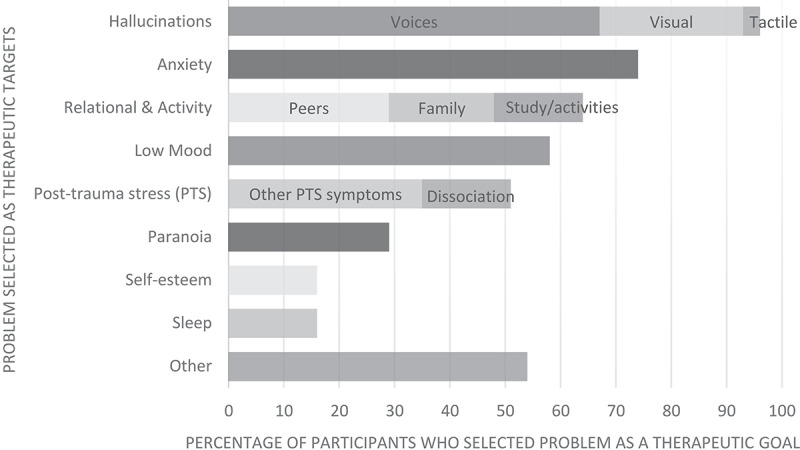
Problems selected as therapeutic targets by young people receiving CBTp as part of the MAPS trial. (Data drawn from 32 adolescents receiving CBTp with 6 trial therapists across 3 research sites).

Optimise engagement by collaboratively identifying meaningful goals that match the young person’s values. MAPS participants highlighted that breaking down their difficulties into tangible, manageable components helped spark interest and alleviated hopelessness that their problems were insurmountable. Rank problems by motivation or prioritise goals that permit early success such as developing coping strategies. Early improvements help build confidence in therapy and their ability to approach more challenging difficulties. Figure 1 contains examples of problems selected as treatment targets as part of the MAPS trial.

“Other” included suicidal thoughts/self-harm (13%), anger/irritability (10%), eating problems (6%), bereavement (3%), and perfectionism (3%).

#### Building engagement

YP are particularly susceptible to public stigma about mental health, potentially increasing concealment of distress and reluctance to seek help. Many YP with FEP will have no or limited prior experiences of mental health services or therapy. The first task is to engage YP and create a safe-enough space for them to engage in what therapy asks of them, and to feel motivated enough to make changes.

Clinicians should explicitly provide an overview of what CBTp will involve and what is required of them, as well as inviting consideration of what they want from therapy. Use clear, relatable language to highlight the structured, collaborative, and goal-focused nature of sessions. It is imperative to be transparent outlining confidentiality and seeking consent about necessary sharing of information if safeguarding concerns arise. This helps avert therapeutic rifts and instils confidence that disclosures will not be unnecessarily shared. Clinicians should offer an interactive therapeutic approach and be flexible with practicalities of appointments (Byrne et al., 2020). Outdoor therapy (with consideration given to confidentiality) can enable greater shared ownership of the therapy space and relationship (Cooley et al., 2020). Over a third of MAPS therapists suggested a longer therapy window would help build engagement and accommodate periods of disengagement, academic examinations, and the impact of risk on therapy (Morrison et al., 2021).

YP new to mental health services may be receptive to information about service provision and psychoeducation about psychosis. Normalising information can powerfully reduce the impact of stigma, potentially helping adolescents feel safer and willing to engage, as noted by Byrne et al. (2020): *I think it’s really good that there was somebody out there who could say actually you know what this is normal, and there’s lots of people who experience it”* (F12, AP+PI)

Within MAPS, adolescents commonly requested age-appropriate information about youth mental health and voice-hearing, particularly recovery stories and personal accounts from other YP, celebrities, or sports players. Whilst existing adult-focused resources can be adapted to improve relevance and appeal to YP; there is a plethora of freely available, well-designed, and informative teen-specific resources which provide accessible and reassuring psychosis-related information (in both written and video format). Examples at the time of writing include Voice Collective; Intervoice; Hearing Voice Network, Understanding Psychosis: A guide for young people and their supporters. Signposting can help avert anxiety caused by unhelpful online searches by this internet-savvy generation. Disseminating information to worried family or friends can boost their ability to support.

#### Involving others: family intervention for psychosis and informal involvement

Clinical guidelines for YP and adults with schizophrenia and psychosis recommend offering FI in conjunction with CBT, whilst acknowledging the limited evidence for YP (National Institute for Health and Clinical Excellence [NICE], 2013, National Collaborating Centre for Mental Health and the National Institute for Health and Care Excellence, 2016). Since many YP live with and receive close support from family members, and peer-influence is high, involving others potentially bolsters therapeutic progress. Byrne et al. (2020) found that despite potential for upset, FI was valued for enabling discussions that improved family members understanding of the young person’s difficulties, in turn facilitating improved communication and support: “*the family session was a chance for me to tell them what I’m going through and a chance for them to tell me what I’ve been doing wrong, and what I’ve been doing right”* (YP06, PI).

Within MAPS, the primary aim of FI was involving family members in supporting the individual’s progress. Early family sessions helped assess the family’s understanding of presenting difficulties and, with the young person’s consent, sharing information about therapeutic goals and formulation. FI goals commonly focused on improving family relations by identifying and changing unhelpful patterns of interaction and communication styles. Families who received MAPS FI reported that receiving recovery-oriented information and signposting to support networks helped promote optimism (Byrne et al., 2020).

Notably, nearly half the YP or their families participating in MAPS were unwilling or unable to commit to optional FI sessions (Morrison et al., 2021). This highlights the need for a flexible approach to involving others in terms of length, location, type of delivery, and ensuring YP hold some autonomy in their treatment decisions.

**Figure 2.**
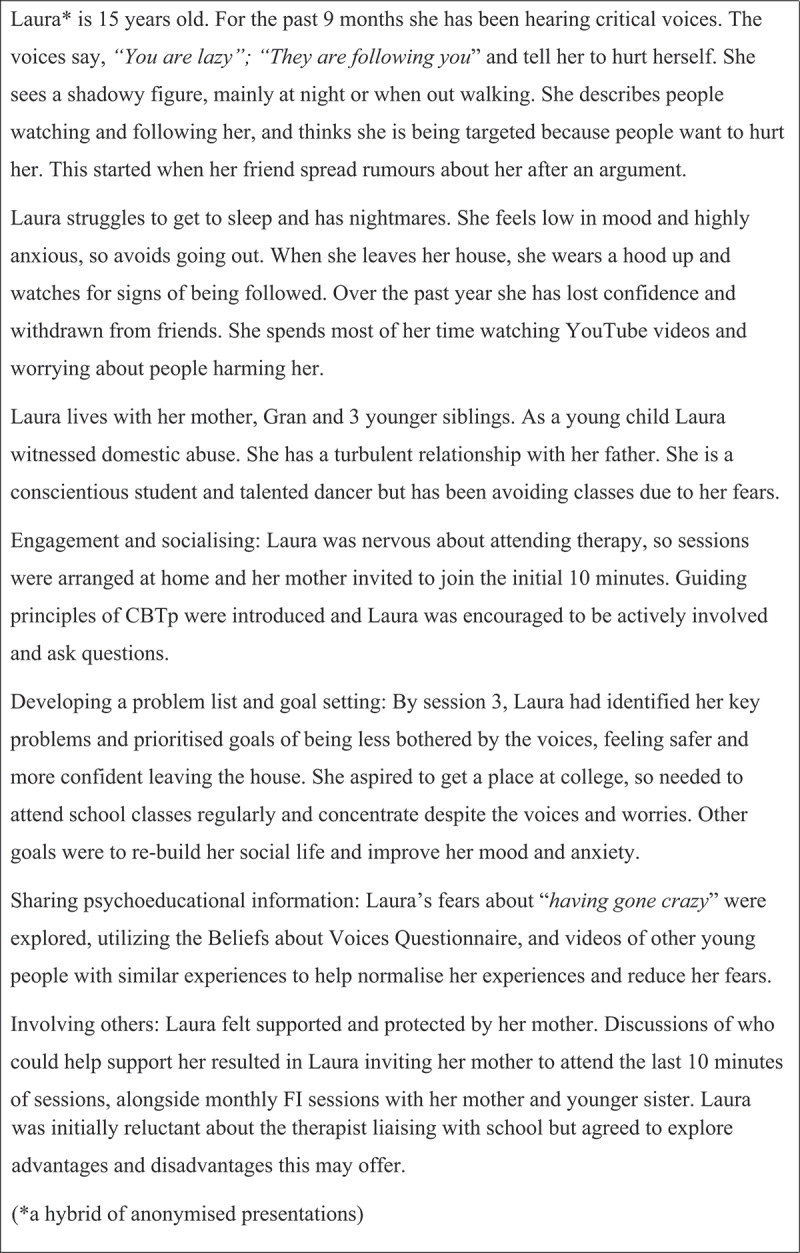
Case example – assessment and engagement.

The decision to involve others or share-specific information with a wider network requires careful consideration and should be openly negotiated with a young person. To invite discussion about wider network, it can be helpful to ask YP where they sleep (rather than where they live). Clinicians can invite collaborative evaluation of potential advantages and disadvantages, considering the balance of desires for inclusion versus privacy. There are many potential benefits of involving others (to supplement CBTp or through FI). Early in therapy, if a young person is ambivalent or nervous, invite family members, carers, partners, or friend to join part of the session. This can build confidence, elicit useful dialogue, and allow sharing of relevant information. Ideally reserve time for one-to-one discussion with the young person to allow disclosure of information they may not wish to share with others (such as drug use, problems at school, domestic abuse). See, Figure 2 for a case example illustrating assessment and engagement.

#### Monitoring and motivating change: use of outcome measures

Validated outcome measures can help assess the presence and severity of symptoms and monitor symptom-related change. Although many standardised questionnaires are validated on norms derived from adult populations, they can be used pragmatically with adolescent populations. Symptom-specific measures can provide concrete means of demonstrating and reflecting on symptom change (such as the revised Beliefs About Voices Questionnaire (BAVQ–R; Chadwick et al., 2000); Voice Power Differential Scale (VPDS; Birchwood et al., 2000); Bird Checklist of Adolescent Paranoia (B-CAP; Bird et al., 2020). Also consider utilising measures for co-morbid problems and social issues such as improving relationships and functioning (e.g. First-Episode Social Functioning Scale for early psychosis; Lecomte et al., 2014).

Fornells‐Ambrojo et al. (2017) found that YP expressed less satisfaction with completing sessional outcome measures compared to other service users. To boost receptiveness, select measures that match the young person’s therapy targets, explain rational for using outcome measures, and seek feedback from the young person about their acceptability and utility. Online or smart-phone delivery may be preferred by YP. MAPS therapists routinely designed idiosyncratic visual analogue scales and ratings of subjective units of distress to monitor change and support developing insight. Feedback noted YP generally found such scales intuitively user-friendly and helpful.

#### Potential challenges encountered working with adolescents experiencing psychosis

Trial therapists encountered a range of challenges specific to working with adolescents which impacted therapeutic progress. These potential barriers, along with suggestions to navigate such challenges, are documented in Table 1.

**Table 1.** Potential barriers to delivering CBTp with adolescents with psychosis and approaches to address these challenges.

Potential barriers (*)	Suggested approaches to navigate challenges
(1) Barriers to engagement	
Engagement may be low, particularly if help-seeking is initiated by others. Adolescents are more susceptible to peer pressure and stigma, typically hold a shorter-term perspective, and strive for independence from adults, so may conceal difficulties. An age-power imbalance between therapist and young person may create a barrier. Some YP may display difficulty communicating or appear distracted (potentially by hearing voices or paranoid ideations) during session.	Agree initial, short-term contract (6-8 sessions) with reviews. Consider offering twice-weekly sessions to initiate early change and bolster engagement. Be flexible with session practicalities. Get out and about together. Arrange between session contacts or send reminders to garner momentum Personalise goals and therapy directly to the young person’s interests/ hobbies Use personalised outcome measures to monitor and demonstrate change Maximise self-efficacy and prioritise true collaboration. Use Socratic and peripheral questioning with genuine curiosity to encourage self-generated discovery. Avoid outright challenging Use age-appropriate language. Routinely invite and address feedback, and work to redress age-power imbalance. Explicitly encourage young person to raise in-session anomalous experiences. Use these as opportunity to elicit info to help understand their experiences. Support young person to experiment with different ways of responding. Ask direct questions such as, “Are you worried that I’m trying to harm you?”. Routinely invite feedback.
(2) Developmental level	
Adolescents will have varying levels of cognitive and emotional maturity and interventions should be tailored accordingly. Some YP may struggle with emotional literacy, difficulty articulating, severe distress, or poor attention.	Creatively scaffold discoveries. Try using : closed concrete questions; experiential examples and age-appropriate metaphors; humour; multi-sensory resources such as diagrams, card-sort tasks, personal accounts, videos, gifs, memes. Utilise smart technology apps and wearable devices To promote emotional literacy use symptom-specific questionnaires to gather information; use visual thermometer charts or emojis to represent degrees of emotion; ask questions employing shifts in time, space and person (e.g. “What would you have thought two years ago? What would your friend think?”); explore imagery Be creative with behavioural experiments to promote hope/enjoyment. Involve others. Troubleshoot barriers Encourage independent problem solving. Use surveys to gather opinions. Conduct role-plays in sessions Use prompts and reflective summaries. Check understanding regularly.
(3) The broader context	
Adolescence can be a turbulent, fast-changing time involving changeable or chaotic living situations. The wider context of family, navigating peer relationships and life events can have significant impact. Issues of intimacy and sexuality may emerge	Remain alert and open to exploring life events. Regularly review formulations and re-visit goals, renegotiating when necessary Balance boundary-setting with offering flexibility. Use agenda-setting to avoid therapeutic drift, whilst allowing time-limited scope to explore life events. If progress in therapy becomes stilted due to firefighting crises, address this explicitly Collaboratively agree practicalities of session arrangements. Consider exam stresses, school or work commitments. Slow pace as required Support development of social skills and normalise stresses of peer relationships. Use problem-solving approach. Where appropriate, liaise with school/other agencies.
(4) Case complexity and symptom severity	
Co-morbid, complex difficulties may severely impact levels of distress and functioning. Common issues for adolescents include difficulties with emotion regulation, sleep, substance misuse, interpersonal difficulties, anti-social behaviour. Working with a vulnerable and complex client group likely has an emotional toll on clinicians.	Use disorder-specific recommended interventions when co-morbid difficulties are the prioritised goal Pay particular attention to assessing and improving sleep - a risk factor for both developing and exacerbating psychotic experiences Look for difficulties relating to emotional regulation and interpersonal difficulties, as well as trauma and dissociation. Introduce affect monitoring, education, and management strategies Where relevant, routinely review and formulate how substance use and anti-social behaviour impact their difficulties and goals. Provide psychoeducation or support a young person to evaluate potential advantages and disadvantages of certain behaviours Consider, formulate, and tailor to (potentially undiagnosed) autistic spectrum traits Use supervision and links with specialist clinical networks to help manage the impact of this work and share resources.

*These potential barriers were identified through clinical practice and supervisory observations, and qualitative findings (Bryne et al., 2020) as part of the MAPS trial, underpinned with reference to neuro-developmental theory (e.g. Blakemore, 2019) and youth work principles (e.g. Fowler et al., 2019).

### CBTp phase 2: formulating maintenance factors and change strategies

Within a CBT approach, formulations encompass key hypotheses about the causes, mechanisms, triggers and maintaining factors contributing to a person’s difficulties. A key objective within CBTp is to evaluate a person’s appraisal or response to their difficulties as helpful or unhelpful. This may lead to modification and motivate action towards identified goals. MAPS trial therapists nearly always developed a shared maintenance formulation by session 3, with around half developing a comprehensive shared longitudinal formulation by session 26 (Morrison et al., 2021).

Formulating style should be adapted to accommodate cognitive and socio-affective capacities. Using the young person’s own language in formulations promotes feeling heard and avoids misunderstandings. Support YP to access key appraisals and associated affective responses through in-vivo experiments or imaginatively designed thought diaries. Consider using smart technology to prompt or record (such as voice memos).

Involving others can provide useful insights. For example, third-party observations can illuminate reactions to aversive situations (such as observing hypervigilant checking, or peers recalling instances where the young person seemed distracted or left early). It may be relevant to incorporate significant others(s) thoughts, feelings, and behaviours into formulations to highlight how their responses may inadvertently maintain difficulties over time (for example, offering reassurance, providing lifts so the young person can continue to avoid public transport). This can lead to generating more helpful ways to support the young person.

**Figure 3.**
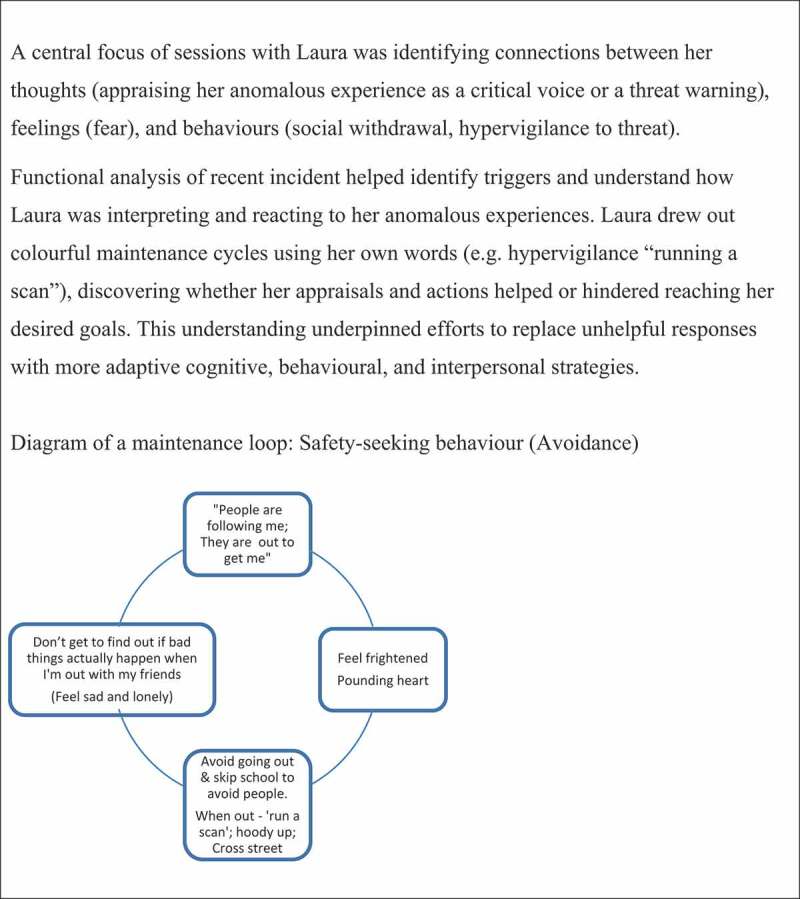
Case example – maintenance formulation.

To develop understanding of likely maintaining factors, clinicians should start simply and scaffold discovery learning. This might involve conducting a simple functional analysis placing triggers, thoughts, feelings, and reactions in columns. Once a young person is familiarised with linking their cognitions, emotions and responses; this framework can be used to identify safety-seeking behaviours (maintenance factors) which act as barriers to achieving their goals. This leads to inform interventions that aim to discover or reinforce more adaptive cognitive, behavioural, and interpersonal tools that promote a sense of control and safety, reduce distress, or increase functioning (in line with goals). This approach can then be replicated to address other maintaining factors, encouraging the young person to take an increasing lead, and generalising learnings beyond therapy. See Figure 3 for case example of developing a maintenance formulation.

**Figure 4.**
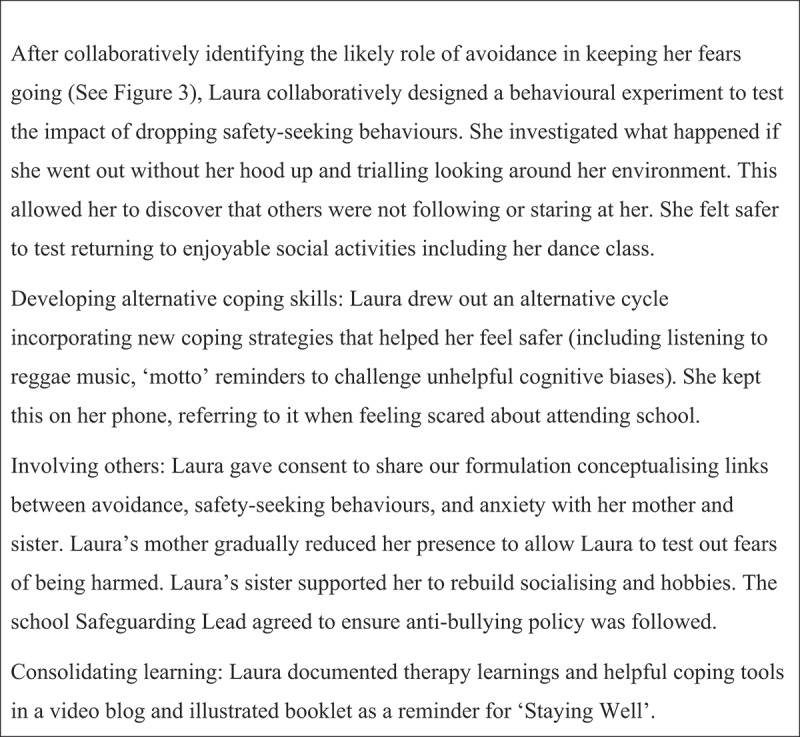
Case example – Change intervention.

The process of assessment and developing a formulation guides the choice and application of interventions to match the needs of a young person. Morrison (2017) details a range of change strategies including cognitive strategies (e.g. evidential analysis), metacognitive strategies (e.g. worry postponement) and behavioural strategies (e.g. behavioural experiments), as well as an emphasis on between-session (“homework”) tasks. See Figure 4 for case example of intervention strategies.

Clinicians may encounter age-specific motivational or systemic barriers to therapeutic change. Adolescents may have limited control over their environment or decision-making (e.g. due to rigid educational schedules, lack of personal space or financial independence). Similarly, systemic factors may also restrict a young person’s repertoire of coping strategies compared to adults (perhaps a partial factor in the greater reliance on cannabis use in teens). For YP who are reluctant to test new adaptive responses, clinicians should use motivational approaches to collaboratively appraise what can feasibly be achieved. Explore perceived risks of change weighted against potential gains, where possible leading to evaluation through a behavioural experiment. Consider support from peers or family, or between-session text encouragement and prompts.

To help overcome ambivalence, the target of change needs to be meaningful and appealing to the YP. Engaging an adolescent via their personal interests (such as gaming, music, art, sports) can be helpful, as can embedding strategies and explanations within these contexts (e.g. “What would your video game character do in this situation?”, “How would an athlete improve a skill?”).

Progress is more likely if active efforts and testing is attempted between sessions. Explaining this rationale to YP can be instrumental in initiating early change. MAPS participants noted that setting between-session tasks for both themself and the therapist emphasised the shared commitment to improve their well-being, and in turn boosted their enthusiasm to complete tasks. Between-session tasks need to be meaningful, relevant, and manageable (and be cautious about using the term “homework”).

Byrne et al. (2020) identified YP found gaining new ways of thinking about and responding to distress particularly helpful: ”*like I will feel something and then I’ll end up going in the same circle of my actions, whereas when someone’s offering you a new way it might change how you do it”* (YP03, AP+PI).

Optimise motivation by ensuring behavioural interventions are developmentally tailored. For example, if working to re-build social connections through graded exposure, consider age-specific expectations about pleasure and success. Creative active-testing approaches can encourage adolescents to develop coping strategies, such as experiential trials of attention training or active-mind techniques to help manage distressing voices i.e. visual, auditory, or physical tasks with a high cognitive load such as reciting song lyrics or poems, skipping, shadow boxing. Other popular tools to manage hallucinations included absorbing mental imagery (e.g. a favourite place evoking positive affect, olfactory distractors with strong, pleasant smells or tastes such as sour candy) or use of immediate sensory feedback (e.g. launching an object through a visual hallucination).

Another key CBTp change strategy that may require tailoring with adolescents is addressing metacognitive beliefs (such as positive beliefs about paranoia, rumination or worry). Teenagers have varying levels of emotional and verbal literacy, dependant on their developmental stage of abstract reasoning, meta-cognition, and social awareness. Bear in mind that people who have experienced developmental trauma tend to have higher rates of memory and attentional difficulties. Some may benefit from greater scaffolding, or utilising apps on smart devices that support development of metacognitive coping strategies such as postponing perseverative rumination. The NHS Apps Library is a useful source of online tools to support mental health and well-being (e.g. “WorryTree” for rumination or “CalmHarm” for managing self-harm ideation). YP living in adversity may benefit from support acquiring problem-solving skills to help damage-limitation or overcome barriers to progressing in therapy.

### CBTp phase 3: longitudinal formulation

**Figure 5.**
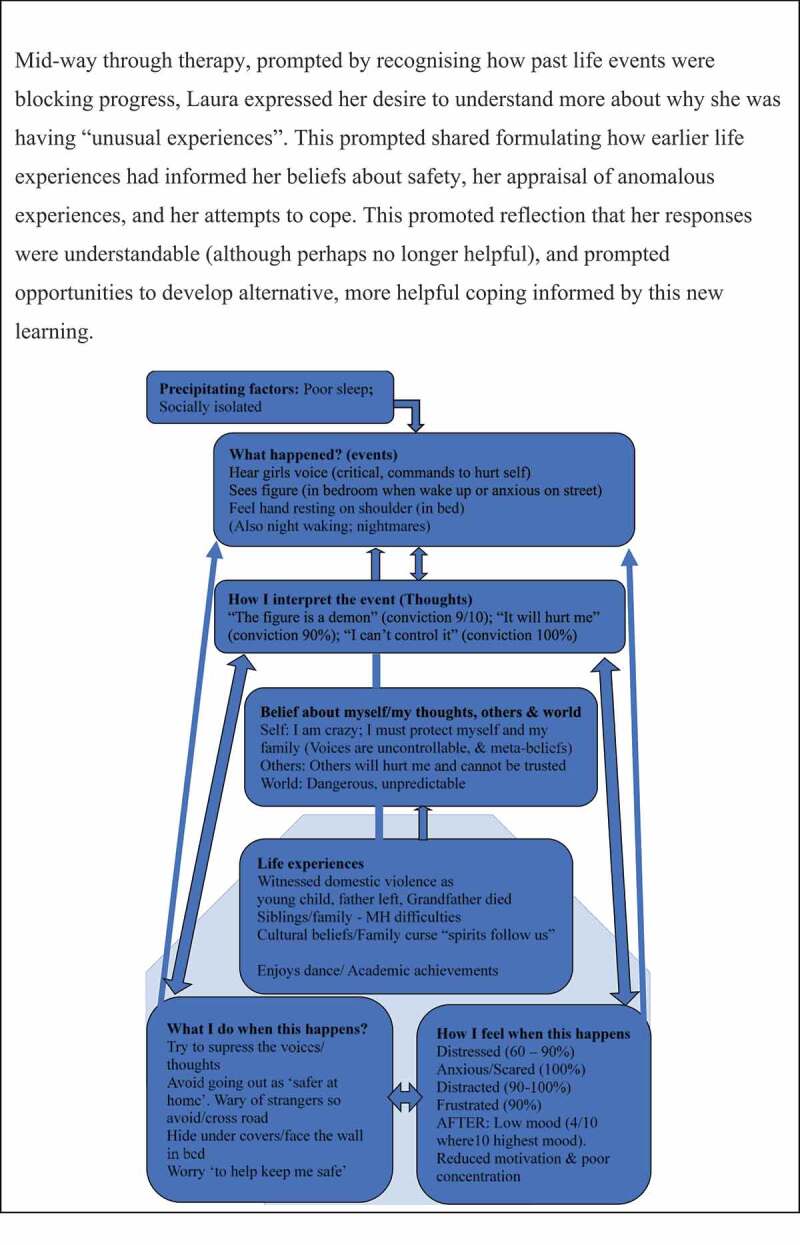
Case example – longitudinal formulation.

Longitudinal formulation is the collaborative process of developing a historical formulation linking life experiences to beliefs formed. Such formulations synthesise complex information into a dynamic understanding that is used to guide therapeutic practices, bridging content between sessions and across the course of therapy. Trial therapists aimed to devise and share a formulation in supervision by session 5. However, sensitivity and care are required to decide the extent and timing of engaging in this as a shared process with YP. Collaboratively developed longitudinal formulations were developed with only around half of the YP, typically around session 16 onwards (after working at maintenance level). Whilst some YP goals remained focused on current coping, others prioritised building their understanding about potential causes of their “unusual experiences”. Shared formulation can be useful if therapy is stuck, or where clear links to trauma exist. Change strategies at this phase may involve schema change methods (Padesky, 1994). It is possible that adolescent core beliefs may be more flexible and amenable to change, since they have been subjected to a shorter period of confirmatory biases in comparison to adults. See Figure 5 for case example of longitudinal formulation.

### CBTp phase 4: consolidation and promoting “staying well”

As with adults, recognising progress is a central aspect of change. Throughout sessions, invite adolescents to reflect and summarise key learnings to help cement understanding and avert relapse. Routinely ask and be responsive to feedback, enquiring if anything important has been missed or could be approached differently. Creative approaches to consolidate young people's learning are crucial. If the YP is distracted or has poor attention, record therapy sessions or create video-summaries of key learnings that can be re-watched and added to. Discuss security of recordings, particularly if sensitive information is disclosed. Consider using technology to set electronic calendar reminders to practice or reinforce more adaptive coping strategies.

Within MAPS, therapy blueprints were developed to support maintenance of gains, with booster sessions offered to facilitate consolidation. Summaries of learnings are more powerful if collaboratively created, and creativity amplifies their memorability and encourages a young person to revisit it; all are central to “staying well”. To aid comprehension and retention, explore the young person’s preferred style of creating a lasting reminder of their progress and coping tools. MAPS participants expressed preference for creative formats, rather than long (unread) reports. Example formats included video blogs summarising the main learnings and action plans to continue progress; text message or bullet-point summaries including drawings, images, memes or mottos; graphic novels, comic strips or cartoon sketches; photographs; letter to future self, and cue-card prompts of helpful coping strategies.

Clinicians should explore who will continue to be involved with supporting a young person to stay well, and help identify a clear, specific outline of what their ongoing supporting role will entail.

## Conclusion

This article describes several approaches for tailoring CBTp assessment, formulation, and interventions to meet the development needs of adolescents with FEP.

Clinical learnings, developed in the context of a methodologically rigorous clinical trial, are shared including techniques to build engagement, involve others, monitor and motivate change, and suggestions of age-relevant resources. Potential challenges that may be encountered working with adolescents experiencing psychosis are highlighted, along with suggestions for addressing these.

Whilst the primary outcome of the MAPS study was to determine feasibility, both quantitative and qualitative findings provide early indications this psychological intervention (CBTp plus FI) delivered either solo or in combination with pharmacological treatment is an acceptable, safe and helpful treatment for YP with first-episode psychosis, seeming to provide benefit in terms of symptoms (Positive and Negative Syndrome Scale total score) and recovery (Questionnaire about the Process of Recovery total). Most YP showed clinical improvement over time, with ten (63%) of 16 participants in the psychological intervention group, five (28%) of 18 in the antipsychotics group, and 11 (65%) of 17 in the combined group showing at least 25% improvement in PANSS total score at 6 months (analysed by intention to treat). There was a high rate of adherence to psychological intervention (defined as six or more sessions of CBT; in 32 [82%] of 39 participants in the monotherapy and combined groups. No findings suggested that psychological interventions in the absence of antipsychotic medication were detrimental (See, Morrison et al., 2020, 2021 for detailed findings).

Participant accounts of CBT in Byrne’s qualitative study highlight largely positive views of interpersonal engagement with therapists, with YP and families viewing CBTp as an interactive approach helpful in building understanding of psychosis and symptom reduction, along with enhancing functioning and coping, although some found it emotionally and cognitively challenging (See, Byrne et al., 2020 for further details).

Importantly the presentations of adolescent psychosis and the cultural context in which they occur are widely variable, so these proposed adaptations are offered as considerations to be personalised for each young person’s presenting needs. To finish on a note of caution, whilst there is a growing evidence base for CBT for adolescents, primarily for anxiety disorders (James et al., 2020), there is limited research exploring the effectiveness of CBTp for adolescents (Anagnostopoulou et al., 2019). Future research is needed to further explore the efficacy and acceptability of CBTp in adolescents, as well as building evidence-based clinical guidance to support and train clinicians to deliver CBTp to meet the developmental needs of each young person.

## Note

1. The MAPS trial aimed to establish feasibility of conducting a large-scale trial to assess efficacy of antipsychotic medication and psychological therapies for adolescents with first-episode psychosis. For details of the trial protocol see Pyle et al. (2019), for findings of the RCT controlled pilot and feasibility study see Morrison et al. (2020); Morrison et al. (2021), and for findings of nested qualitative interviews with young people and family see Byrne et al. (2020).

## References

[cit0001] Anagnostopoulou, N., Kyriakopoulos, M., & Alba, A. (2019). Psychological interventions in psychosis in children and adolescents: A systematic review. *European Child & Adolescent Psychiatry*, 28(6), 735–746. 10.1007/s00787-018-1159-3 29728871

[cit0002] Birchwood, M., Meaden, A., Trower, P., Gilbert, P., & Plaistow, J. (2000). The power and omnipotence of voices: Subordination and entrapment by voices and significant others. *Psychological Medicine*, 30(2), 337–344. 10.1017/S0033291799001828 10824654

[cit0003] Bird, J. C., Loe, B. S., Kirkham, M., Fergusson, E. C., Shearn, C., Stratford, H., Teale, A.-L., Waite, F., & Freeman, D. (2020). The assessment of paranoia in young people: Item and test properties of the bird checklist of adolescent paranoia. *Schizophrenia Research*, 220, 116–122. 10.1016/j.schres.2020.03.046 32247744

[cit0004] Blackburn, I. M., James, I. A., Milne, D. L., Baker, C., Standart, S., Garland, A., & Reichelt, F. K. (2001). The revised cognitive therapy scale (CTS-R): Psychometric properties. *Behavioural and Cognitive Psychotherapy*, 29(4), 431. 10.1017/S1352465801004040

[cit0005] Blakemore, S. J. (2019). Adolescence and mental health. *The Lancet*, 393(10185), 2030–2031. 10.1016/S0140-6736(19)31013-X 31106741

[cit0006] Boeing, L., Murray, V., Pelosi, A., McCabe, R., Blackwood, D., & Wrate, R. (2007). Adolescent-onset psychosis: Prevalence, needs and service provision. *The British Journal of Psychiatry*, 190(1), 18–26. 10.1192/bjp.190.1.18 17197652

[cit0007] Browning, S., Corrigall, R., Garety, P., Emsley, R., & Jolley, S. (2013). Psychological interventions for adolescent psychosis: A pilot-controlled trial in routine care. *European Psychiatry*, 28(7), 423–426. 10.1016/j.eurpsy.2013.05.008 23968892

[cit0008] Buckley, P. F., Miller, B. J., Lehrer, D. S., & Castle, D. J. (2009). Psychiatric comorbidities and schizophrenia. *Schizophrenia Bulletin*, 35(2), 383–402. 10.1093/schbul/sbn135 19011234 PMC2659306

[cit0009] Byrne, R. E., Bird, J. C., Reeve, S., Jones, W., Shiers, D., Morrison, A. P., Pyle, M., & Peters, S. (2020). Understanding young people’s and family members’ views of treatment for first episode psychosis in a randomised controlled trial (MAPS). *EClinicalMedicine*, 24 (7) , 100417. 10.1016/j.eclinm.2020.100417 32775967 PMC7393652

[cit0010] Chadwick, P., Lees, S., & Birchwood, M. A. X. (2000). The revised beliefs about voices questionnaire (BAVQ–R). *The British Journal of Psychiatry*, 177(3), 229–232. 10.1192/bjp.177.3.229 11040883

[cit0011] Cooley, S. J., Jones, C. R., Kurtz, A., & Robertson, N. (2020). ‘Into the Wild’: A meta-synthesis of talking therapy in natural outdoor spaces. *Clinical Psychology Review*, 77, 101841. 10.1016/j.cpr.2020.101841 32179342

[cit0012] Díaz-Caneja, C. M., Pina-Camacho, L., Rodríguez-Quiroga, A., Fraguas, D., Parellada, M., & Arango, C. (2015). Predictors of outcome in early-onset psychosis: A systematic review. *Schizophrenia*, 1(1), 1–10. doi:10.1038/npjschz.2014.5 PMC4849440 27336027

[cit0013] Fornells‐Ambrojo, M., Johns, L., Onwumere, J., Garety, P., Milosh, C., Iredale, C., Peters, E., Webster, A., & Jolley, S. (2017). Experiences of outcome monitoring in service users with psychosis: findings from an improving access to psychological therapies for people with severe mental illness (IAPT‐SMI) demonstration site. *British Journal of Clinical Psychology*, 56(3), 253–272. 10.1111/bjc.12136 28493592

[cit0014] Fowler, D., Hodgekins, J., Berry, C., Clarke, T., Palmier-Claus, J., Sacadura, C., Graham, A., Lowen, C., Steele, A., Pugh, K., Fraser, S., Fitzsimmons, M., & French, P. Social recovery therapy: A treatment manual. (2019). *Psychosis*, 11(3), 261–272. 1752-2439. 10.1080/17522439.2019.1607891

[cit0015] Haddock, G., Lewis, S., Bentall, R., Dunn, G., Drake, R., & Tarrier, N. (2006). Influence of age on outcome of psychological treatments in first-episode psychosis. *The British Journal of Psychiatry*, 188(3), 250–254. 10.1192/bjp.188.3.250 16507967

[cit0016] James, A. C., Reardon, T., Soler, A., James, G., & Creswell, C. (2020). Cognitive behavioural therapy for anxiety disorders in children and adolescents. *Cochrane Database of Systematic Reviews* 11(11). doi:10.1002/14651858.CD013162.pub2 PMC8092480 33196111

[cit0017] Jolley, S., Kuipers, E., Stewart, C., Browning, S., Bracegirdle, K., Basit, N., Gin, K., Hirsch, C., Corrigall, R., Banerjea, P., Turley, G., Stahl, D., & Laurens, K. R. (2018). The Coping with Unusual Experiences for Children Study (CUES): A pilot randomized controlled evaluation of the acceptability and potential clinical utility of a cognitive behavioural intervention package for young people aged 8–14 years with unusual experiences and emotional symptoms. *British Journal of Clinical Psychology*, 57(3), 328–350 10.1111/bjc.12176. 29527754

[cit0018] Jones, P. B. (2013). Adult mental health disorders and their age at onset. *The British Journal of Psychiatry*, 202(s54), s5–s10. 10.1192/bjp.bp.112.119164 23288502

[cit0019] Lecomte, T., Corbière, M., Ehmann, T., Addington, J., Abdel-Baki, A., & MacEwan, B. (2014). Development and validation of the first episode social functioning scale. *Psychiatry Research*, 216(3), 412–417. 10.1016/j.psychres.2014.01.044 24613006

[cit0020] Morrison, A. P., Pyle, M., Byrne, R., Broome, M., Freeman, D., Johns, L., James, A., Husain, N., Whale, R., MacLennan, G., Norrie, J., Hudson, J., Peters, S., Davies, L., Bowe, S., Smith, J., Shiers, D., Joyce, E., Jones, W., … Maughan, D. (2021). Psychological intervention, antipsychotic medication or a combined treatment for adolescents with a first episode of psychosis: The MAPS feasibility three-arm RCT. *Health Technology Assessment (Winchester, England)*, 25(4), 1. 10.3310/hta25040 PMC7869006 33496261

[cit0021] Morrison, A. P., Pyle, M., Maughan, D., Johns, L., Freeman, D., Broome, M. R., Husain, N., Fowler, D., Hudson, J., MacLennan, G., Norrie, J., Shiers, D., Hollis, C., James, A., Morrison, A. P., Pyle, M., Maughan, D., Johns, L., Freeman, D., … James, A. (2020). Antipsychotic medication versus psychological intervention versus a combination of both in adolescents with first-episode psychosis (MAPS): A multicentre, three-arm, randomised controlled pilot and feasibility study. *The Lancet Psychiatry*, 7(9), 788–800. 10.1016/S2215-0366(20)30248-0 32649925 PMC7606914

[cit0022] Morrison, A. P. (2017). A manualised treatment protocol to guide delivery of evidence- based cognitive therapy for people with distressing psychosis: Learning from clinical trials. *Psychosis*, 9(3), 271–281. 10.1080/17522439.2017.1295098

[cit0023] National Collaborating Centre for Mental Health (NCCMH). (2014). *Psychosis and schizophrenia in adults: The NICE guideline on treatment and management (NICE clinical guideline 178)*. NICE.

[cit0024] National Collaborating Centre for Mental Health and the National Institute for Health and Care Excellence. (2016). *Implementing the early intervention in psychosis access and waiting time standard: Guidance*. NICE.

[cit0025] National Institute for Health and Clinical Excellence (NICE). (2013). *Psychosis and Schizophrenia in children and young people: Recognition and management*. 32186837

[cit0026] Padesky, C. A. (1994). Schema change processes in cognitive therapy. *Clinical Psychology & Psychotherapy*, 1(5), 267–278. 10.1002/cpp.5640010502

[cit0027] Patel, M., Chawla, R., Krynicki, C. R., Rankin, P., & Upthegrove, R. (2014). Health beliefs and carer burden in first episode psychosis. *BMC Psychiatry*, 14(1), 1–7. 10.1186/1471-244X-14-171 PMC4094457 24913656

[cit0028] Pyle, M., Broome, M. R., Joyce, E., MacLennan, G., Norrie, J., Freeman, D., Fowler, D., Haddad, P. M., Shiers, D., Hollis, C., Smith, J., Liew, A., Byrne, R. E., French, P., Peters, S., Hudson, J., Davies, L., Emsley, R., Yung, A., … Morrison, A. P. (2019). Study protocol for a randomised controlled trial of CBT vs antipsychotics vs both in 14–18-year-olds: Managing Adolescent first episode psychosis: A feasibility study (MAPS). *Trials*, 20(1), 1–13. 10.1186/s13063-019-3506-1 31272477 PMC6611021

[cit0029] Radez, J., Reardon, T., Creswell, C., Lawrence, P. J., Evdoka-Burton, G., & Waite, P. (2020). Why do children and adolescents (not) seek and access professional help for their mental health problems? A systematic review of quantitative and qualitative studies. *European Child & Adolescent Psychiatry*, 30, 1–29. 10.1007/s00787-019-01469-4 PMC7932953 31965309

[cit0030] Singh, S. P., Brown, L., Winsper, C., Gajwani, R., Islam, Z., Jasani, R., Parsons, H., Rabbie-Khan, F., & Birchwood, M. (2015). Ethnicity and pathways to care during first episode psychosis: The role of cultural illness attributions. *BMC Psychiatry*, 15(1), 287. 10.1186/s12888-015-0665-9 26573297 PMC4647639

